# Distinct RNA profiles in subpopulations of extracellular vesicles: apoptotic bodies, microvesicles and exosomes

**DOI:** 10.3402/jev.v2i0.20677

**Published:** 2013-09-12

**Authors:** Rossella Crescitelli, Cecilia Lässer, Tamas G. Szabó, Agnes Kittel, Maria Eldh, Irma Dianzani, Edit I. Buzás, Jan Lötvall

**Affiliations:** 1 Department of Internal Medicine and Clinical Nutrition, Krefting Research Centre, University of Gothenburg, Gothenburg, Sweden; 2 Department of Health Sciences, University of Eastern Piedmont, Novara, Italy; 3 Department of Genetics, Cell and Immunobiology, Semmelweis University, Budapest, Hungary; 4 Institute of Experimental Medicine, Hungarian Academy of Sciences, Budapest, Hungary

**Keywords:** apoptotic bodies, microvesicles, exosomes, extracellular vesicles, ultracentrifugation, characterization, RNA, electron microscopy

## Abstract

**Introduction:**

In recent years, there has been an exponential increase in the number of studies aiming to understand the biology of exosomes, as well as other extracellular vesicles. However, classification of membrane vesicles and the appropriate protocols for their isolation are still under intense discussion and investigation. When isolating vesicles, it is crucial to use systems that are able to separate them, to avoid cross-contamination.

**Method:**

EVs released from three different kinds of cell lines: HMC-1, TF-1 and BV-2 were isolated using two centrifugation-based protocols. In protocol 1, apoptotic bodies were collected at 2,000×g, followed by filtering the supernatant through 0.8 µm pores and pelleting of microvesicles at 12,200×g. In protocol 2, apoptotic bodies and microvesicles were collected together at 16,500×g, followed by filtering of the supernatant through 0.2 µm pores and pelleting of exosomes at 120,000×g. Extracellular vesicles were analyzed by transmission electron microscopy, flow cytometry and the RNA profiles were investigated using a Bioanalyzer^®^.

**Results:**

RNA profiles showed that ribosomal RNA was primary detectable in apoptotic bodies and smaller RNAs without prominent ribosomal RNA peaks in exosomes. In contrast, microvesicles contained little or no RNA except for microvesicles collected from TF-1 cell cultures. The different vesicle pellets showed highly different distribution of size, shape and electron density with typical apoptotic body, microvesicle and exosome characteristics when analyzed by transmission electron microscopy. Flow cytometry revealed the presence of CD63 and CD81 in all vesicles investigated, as well as CD9 except in the TF-1-derived vesicles, as these cells do not express CD9.

**Conclusions:**

Our results demonstrate that centrifugation-based protocols are simple and fast systems to distinguish subpopulations of extracellular vesicles. Different vesicles show different RNA profiles and morphological characteristics, but they are indistinguishable using CD63-coated beads for flow cytometry analysis.

Extracellular vesicles (EVs) are membranous vesicles naturally released by most cells (1–9). EVs can be broadly classified into three main classes, based primarily on their size and presumed biogenetic pathways: (a) apoptotic bodies (ABs), 800–5,000 nm diameter and released by cells undergoing programmed cell death, (b) microvesicles (MVs), also referred to as shedding MVs, are large membranous vesicles (50–1,000 nm diameter) that are produced by budding from the plasma membrane (c) and finally exosomes (EXOs), 40–100 nm diameter vesicles considered to be of endocytic origin (10, 11).

Despite some presumed distinct features, numerous similarities exist among the different EVs with respect to their physical characteristics and biochemical composition (12–15), which make the separation of different subsets challenging (12). Because of their small size, many EVs are below the detection range of conventional detection methods such as light microscopy. Consequently, recovery and contamination among vesicles in the separation process cannot be reliably controlled. Furthermore, isolation protocols and the nomenclature are not fully standardized in the field at this point. In most studies, vesicles are isolated by differential centrifugation steps which are considered to be the “golden standard” to isolate different types of EVs (16). Differential centrifugation involves multiple sequential centrifugations, each time removing the pellet and the supernatant, and includes increasing the centrifugal force to isolate smaller and less dense components in the subsequent steps. In general, centrifugal force at 200–1,500×g are used to pellet cells and “cellular debris,” 10,000–20,000×g to pellet vesicles with a size between 100 and 800 nm (generally called MVs) and between 100,000 and 200,000×g to pellet the smallest vesicles with a diameter <100 nm (generally referred to as EXOs) (17).

Besides the size and density of vesicles, the efficiency to isolate vesicles depends on the shape and viscosity of the solution, as well as on temperature, centrifugation time and the type of rotor used for the centrifugation (fixed-angle rotor or swinging buckets). As vesicles are heterogeneous, complete separation of vesicles with a certain diameter and/or density is still unlikely with this approach. Besides differential centrifugations, filtration has also been applied to remove larger vesicles from smaller ones. Although the pore size of filters is often well defined, increasing forces have to be applied with decreasing pore size, which can result in artefacts (12, 17).

Although flow cytometry and Western blot has been utilized to identify and characterize nano-sized vesicles (18), the golden standard remains to be transmission electron microscopy (TEM) (19), which is the only method by which both the size and morphology of the isolated vesicles can be determined simultaneously (12). Attempts to separate different vesicles to allow analysis of their diverse functions and description of their different contents also remain crucial for the development of the field.

In this study, we have used differential centrifugation steps to achieve a relative separation of ABs, MVs and EXOs from several different cell lines, with the hypothesis that the RNA profiles are different in different types of vesicles, but similar among vesicles from different types of cells. To do this, three fundamentally different cell lines were cultured *in vitro*, including a human mast cell line (HMC-1), a human erythroleukemia cell line (TF-1) and a mouse microglia cell line (BV-2). Different EVs were isolated to determine their respective RNA profiles. To determine the morphology of the different vesicles, the subpopulations of EVs from the different cells were visualized using TEM of sectioned vesicle pellets.

## Materials and methods

### Cell cultures

The HMC-1 (J. Butterfield, Mayo Clinic, Rochester, MN, USA) used in our earlier studies (20, 21) was cultured in IMDM (Sigma-Aldrich, St. Louis, MO, USA) containing 10% foetal bovine serum (FBS, Sigma-Aldrich), 100 U ml^−1^ penicillin, 100 µg ml^−1^ streptomycin, 2 mM l-glutamine and 1.2 mM α-thioglycerol (Sigma-Aldrich). The cytokine-dependent erythroleukemia cell line TF-1 (ATCC number: CRL-2003) was grown in RPMI 1640 medium supplemented with 10% FBS, 100 U ml^−1^ penicillin, 100 µg ml^−1^ streptomycin, 2 mM l-glutamine (all reagents were from Sigma-Aldrich) and 5 ng ml^−1^ GM-CSF (granulocyte-macrophage colony-stimulating factor, Miltenyi Biotec, Lund, Sweden). The BV-2 murine microglia cells were grown in RPMI supplemented by 10% FBS (Gibco Invitrogen Corporation, Carlsbad, CA, USA) and 4 µg ml^−1^ ciprofloxacin (Fresenius Kabi Deutschland GmbH, Bad Homburg v.d.H, Germany). For all FBS used in the cell cultures, pure foetal bovine serum was depleted from EXOs prior to use, by ultracentrifugation at 120,000×g for 18 hours, using a Ti45 rotor (Beckman Coulter, Brea, CA, USA). Cell viability was assessed using trypan blue exclusion methods.

### Vesicle isolation

**Fig. 1:**
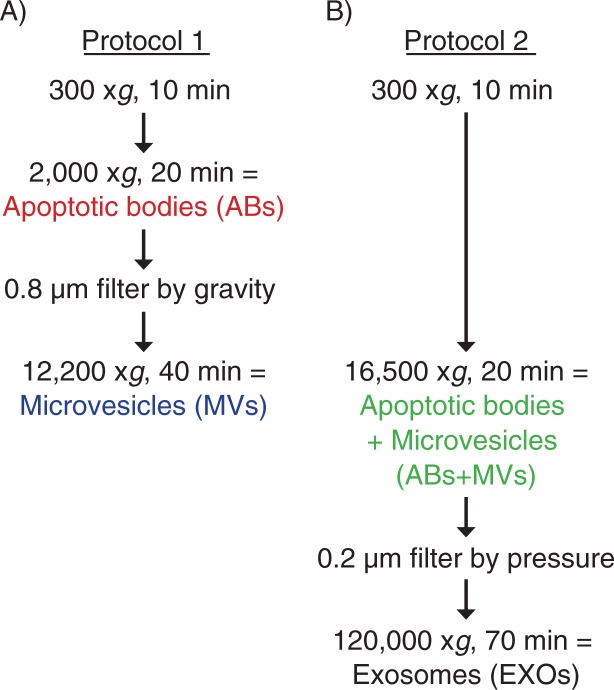
Flow chart over two different differential centrifugation-based protocols. Apoptotic bodies (ABs) and microvesicles (MVs) were isolated separately using protocol 1 (A). ABs and MVs were isolated together (ABs+MVs) followed by exosome (EXO) isolation, using protocol 2 (B).

Vesicles were prepared from the supernatant of HMC-1, TF-1 and BV-2 cells (1–2×10^6^ cells ml^−1^) using two different centrifugation-based protocols. Briefly, for both protocols, cells were isolated and removed by pelleting with centrifugation at 300×g for 10 minutes. Vesicles were then collected from the supernatant through differential centrifugation steps (Fig. 1).

Protocol 1: ABs and MVs were isolated by differential centrifugations and micro-filtration as previously described (22). The supernatant harvested from the cells was centrifuged at 2,000×g for 20 minutes to collect ABs. This supernatant was then filtered by gravity through 0.8 µm filters (GE healthcare, Whatman^®^, UK) to remove particles >800 nm. The supernatant was again collected and further used to isolate MVs. MVs were pelleted by centrifugation at 12,200×g for 40 minutes (Fig. 1A). All centrifugation steps in both protocols 1 and 2 were performed at 4°C.

Protocol 2: ABs+MVs and EXOs were isolated by differential centrifugations and nano-filtration as previously described (21). ABs+MVs were collected together in this protocol by a centrifugation of the cell supernatant at 16,500×g for 20 minutes. The supernatant from this step was filtered through 0.2 µm filters (with gentle pressure) (Sarstedt, Nümbrecht-Rommelsdorf, Germany) to remove particles larger than 200 nm. EXOs were then pelleted by ultracentrifugation at 120,000×g for 70 minutes (Fig. 1B).

### RNA isolation and detection

RNA was isolated from vesicles (n=4 for HMC-1 and TF-1 cells and n=3 for BV-2 cells) using miRCURY™ RNA Isolation Kit (Exiqon, Vedbaek, Denmark) according to the manufacturer's protocol. Detection, quality, yield and size of the vesicular RNA were analyzed using capillary electrophoresis (Agilent RNA 6000 Nano Kit on an Agilent 2100 Bioanalyzer^®^, Agilent Technologies, Santa Clara, CA, USA). One microlitre RNA in solution was analyzed according to the manufacturer's protocol as previously described (23).

### Induction and determination of apoptosis

To induce apoptosis, TF1 cells were incubated with 100 ng ml^−1^ of recombinant human TNF-related apoptosis-inducing ligand (TRAIL; PeproTech Inc., Rocky Hill, NJ, USA). Apoptosis was assessed after 2, 4, 8, 24 and 48 hours by using PE Annexin V Apoptosis Detection Kit I (BD-Pharmingen™, San Jose, CA, USA) according to the manufacturer's protocol. Briefly, after two washes in cold PBS, cells were resuspended in 1× binding buffer (10×: 0.1 M Hepes/NaOH (pH 7.4) 1.4 M NaCl, 25 mM CaCl_2_) at 1×10^6^ cells ml^−1^. One hundred microlitres of cellular suspension was transferred in a FACS tube and 5 µl of Annexin V-PE antibody and 5 µl of the vital dye 7-Amino-Actinomycin (7-AAD) were added. Cells with intact membranes exclude 7-AAD, whereas the membrane of necrotic cells is permeable to 7-AAD. Apoptotic cells are identified by positivity for Annexin-V. After 15 minutes of incubation at room temperature (RT) in the dark, 400 µl of 1× binding buffer was added and the fluorescence was determined by a FACSAria (BD Bio-sciences, San Jose, CA, USA). The flow cytometry data were analyzed using the FlowJo Software (Tri Star Inc., Ashland, OR, USA) (n=2). ABs were collected after 4, 24 and 48 hours of TRAIL treatment, while the other populations of EVs (MVs, AB+MVs and EXOs) were collected after 48 hours only. The RNA profiles were analyzed in all samples as described above (n=2).

### Transmission electron microscopy

The vesicular pellets obtained by the two differential centrifugation-based protocols were submitted to TEM. Briefly, after isolation (see “vesicle isolation” section) pellets were fixed at 4°C overnight. The fixative contained 4% paraformaldehyde in 0.01 M phosphate buffer with pH 7.4 (filtered through 0.22 µm filters). After washing with PBS, the preparations were post-fixed in 1% OsO_4_ (Taab Laboratories Equipment Ltd., Aldermaston, England, UK) for 30 minutes. After rinsing with distilled water, the pellets were dehydrated in graded ethanol, including block staining with 1% uranyl-acetate in 50% ethanol for 30 minutes, and embedded in Taab 812 (Taab). After overnight polymerization at 60°C and sectioning for TEM, the ultrathin sections were analyzed with a Hitachi 7100 electron microscope equipped by Megaview II (lower resolution, Soft Imaging System) digital camera.

### Flow cytometry of vesicles

The protein concentration of the vesicle preparations was measured using the BCA™ Protein Assay Kit (Pierce, Thermo Scientific, Rockford, IL, USA). Antibody-coated beads were prepared as previously described (20, 24). Briefly, for the immune-isolation, 4-µm-diameter aldehyde/sulfate latex beads (Interfacial Dynamics, Life Technologies, Carlsbad, CA, USA) were incubated with 12.5 µg purified anti-CD63 antibody (clone H5C6, BD Biosciences), with the same volume of MES buffer under gentle agitation at RT overnight.

Vesicles (20 µg) were resuspended in PBS and loaded onto the anti-CD63-coated beads (6×10^4^) and were incubated overnight at 4°C under agitation. Vesicle-coated beads were incubated for 30 minutes with 100 mM glycine to block remaining binding sites. The bead–vesicle complexes were washed twice in PBS with 3% FBS (prior ultracentrifuged at 120,000×g for 18 hours). The bead–vesicle complexes were resuspended in IgG (Sigma-Aldrich) and incubated for 15 minutes at RT, before being washed twice more. The tetraspanins CD9, CD63 and CD81, known to be enriched in EXOs, were investigated for its presence on the vesicles. The bead–vesicle complexes were incubated with PE-labelled anti-CD9 (clone M-L13), anti-CD63 (clone H5C6, the same antibody as used to coat the beads), anti-CD81 (clone JS-81) or the corresponding isotype control (all antibodies were from BD Biosciences) for 40 minutes at RT under agitation, washed twice and then acquired by a FACSAria (BD Biosciences) (n=3). The flow cytometry data were analyzed using the FlowJo Software (Tri Star Inc., Ashland, OR, USA).

## Results

### Subpopulations of EVs harbour different RNA profiles

**Fig. 2:**
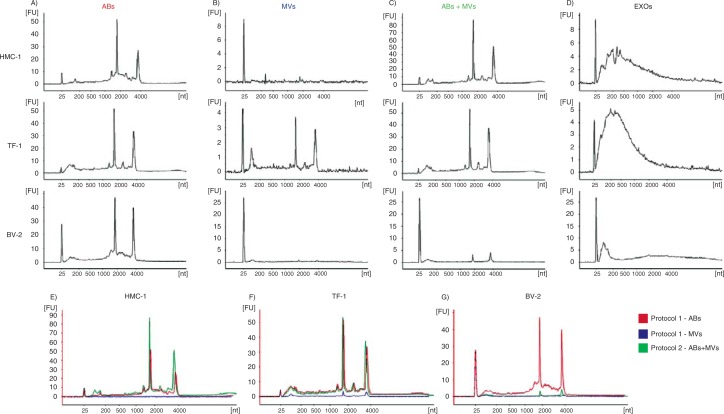
RNA profiles from different subpopulations of extracellular vesicles (EVs). RNA was extracted from vesicles released by three different cell lines; HMC-1 (human mast cell line), TF-1 (human erythroleukemia cells) and BV-2 (mouse microglia cells). The electropherograms show the size distribution in nucleotides (nt) and fluorescence intensity (FU) of total RNA in apoptotic bodies (ABs), microvesicles (MVs), ABs and MVs together (ABs+MVs) and exosomes (EXOs). The short peak at 25 nt is an internal standard. (A) In ABs the most dominant peaks are the 18S and 28S ribosomal RNA. (B) The 18S and 28S rRNA are not evident in MVs from HMC-1 and BV-2, but only obvious in MVs from TF-1, however at low concentrations. (C) 18S and 28S peaks are evident in the pellet composed by ABs and MVs together (ABs+MVs). (D) In EXOs small RNA is dominating, with no or very small rRNA peaks detected. (E–G) The overlapping profiles from ABs (in red) and MVs (in blue) and both collected together (ABs+MVs − in green), suggesting that the contribution of 18S and 28S rRNA is by ABs. The electropherograms are representative of n=4.

RNA profiles in the different vesicular fractions from the three cell lines, HMC-1, TF-1 and BV-2 were analyzed using a Bioanalyzer^®^. Using the vesicle isolation protocols described in Fig. 1, different RNA profiles were observed in the vesicular fractions considered to harbour ABs, MVs and EXOs (Fig. 2A–D). Thus, the technique reveals two dominant peaks, corresponding to the ribosomal RNA (rRNA) subunits 18S and 28S, in ABs from HMC-1, TF-1 and BV-2 cells (Fig. 2A). The rRNA peaks were lacking or were very low in MVs from HMC-1 and BV-2 cells, but could be observed in MVs from TF-1 cells (Fig. 2B). In the 16,500×g pellet, which contained both ABs and MVs, similar RNA profiles as seen for ABs were observed in vesicles from all cell lines (Fig. 2C). In EXOs, the RNA profile from all three cell lines lacked the rRNA peaks, but showed the presence of small RNAs (Fig. 2D). Bioanalyzer^®^ RNA profiles from the two different protocols are also illustrated in the same figure from each cell line (Fig. 2E–G). These data argue that the rRNA peaks are mainly contributed by the ABs and not by MVs.

**Fig. 3:**
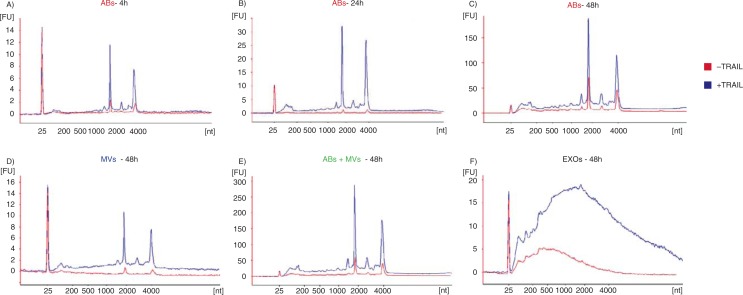
RNA profiles from different subpopulations of extracellular vesicles (EVs) after TRAIL-induced apoptosis. The electropherograms show the RNA size distribution in nucleotides (nt) and fluorescence intensity (FU) in apoptotic bodies (ABs), microvesicles (MVs), ABs and MVs together (ABs+MVs) and exosomes (EXOs) in TF-1 cells with and without TRAIL treatment. The short peak at 25 nt is an internal standard. (A–C) RNA profiles from ABs released by TF-1 cells after 4, 24, 48 hours of TRAIL treatment (in blue) and without TRAIL (in red). After 4 hours (A), 24 hours (B) and 48 hours (C) of TRAIL treatment, in ABs, the peaks of 18S and 28S rRNAs are more prominent comparing with ABs released in the absence of TRAIL. (D–F) RNA profiles from MVs, ABs+MVs and EXOs released by TF-1 cells after 48 hours of TRAIL treatment (in blue) and without TRAIL (in red). (D) The low 18S and 28S rRNA peaks in MVs without TRAIL (in red) become much more prominent after TRAIL treatment (in blue). (E) The highest rRNA peaks are seen in the pellet composed by ABs and MVs together (ABs+MVs). (F) After 48 hours of TRAIL-induced apoptosis, increased amount of small RNAs is observed in exosomes (EXOs). The electropherograms are representative of n=2.

To better understand whether the ABs collected at 2,000×g (protocol 1, Fig. 1) were indeed representative of cells undergoing programmed cell death, recombinant human TRAIL was used to induce apoptosis in the TF-1 cells, as TRAIL has previously been shown to induce apoptosis (25–27). Apoptosis and primary/secondary necrosis were assessed after 2, 4, 8, 24 and 48 hours by flow cytometry using Annexin V-PE and 7-AAD staining, respectively. Upon induction of apoptosis by TRAIL, a three-fold increase in the proportion of apoptotic cells was detected at both 2 and 4 hours without any increase in the ratio of necrotic cells (data not shown). The percentage of apoptotic cells reached 60.2% by 48 hours. However, from 8 hours there was also an increase in the ratio of necrotic cells (reaching 9.7 and 22.6% by 24 and 48 hours, respectively). ABs were collected at 4 hours (when apoptosis was induced without any necrosis), at 24 hours (when cells showed apoptosis with moderate (<10%) necrosis) and at 48 hours (when cells showed increased necrosis and apoptosis but with three times as much apoptosis). The effect of apoptosis on the RNA content in MVs and EXOs were also determined, but due to low-yield MVs and EXOs were only collected and analyzed at 48 hours. Figure 3 shows RNA profiles in vesicles collected with/without TRAIL treatment. As expected, after induction of apoptosis by TRAIL, the amount of rRNA in vesicular fractions increased, suggesting increased amounts of ABs (Fig. 3A–C). In MVs, prominent rRNA peaks were present after TRAIL treatment. The increased quantities of rRNA could also be explained by an increased presence of ABs in this vesicular fraction (Fig. 3D).

Larger amount of rRNA was observed in the pellet composed by ABs and MVs after TRAIL treatment compared to the pellet obtained without TRAIL treatment (Fig. 3E). In the EXO fraction, the RNA profiles were similar under either condition, but a greater quantity of RNAs was evident in the EXO pellet collected after TRAIL treatment (Fig. 3F).

**Fig. 4:**
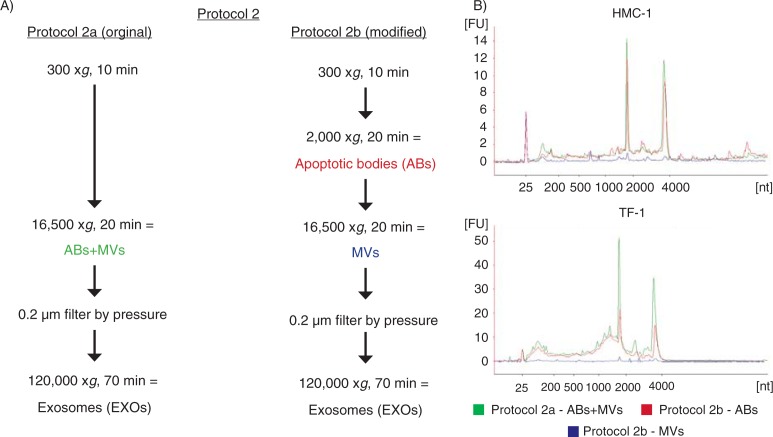
Flow chart over the original and modified protocol 2. (A) In the modification of protocol 2, a 2,000×g step was added to isolate apoptotic bodies (ABs) and microvesicles (MVs) separately, prior to EXOs isolation (here called protocol 2b). (B) The RNA profiles from the different subpopulation of extracellular vesicles (EVs) collected using protocol 2a and 2b. RNA was extracted from vesicles releases from two different cell lines; HMC-1 and TF-1. Shown here are the overlapping profiles from ABs (ABs – in red), MVs (MVs – in blue) and both of them collected together (ABs+MVs − in green), indicating that the contribution of 18S and 28S rRNA is primarily by ABs. The electopherograms show the size distribution in nucleotides (nt) and fluorescence intensity (FU) of total RNA. The peak at 25 nt is an internal standard. The electropherograms are representative of n=3.

To verify if the contribution to the rRNA (18S and 28S subunits) in the ABs+MVs pellet was provided by ABs and not by MVs, a modification of protocol 2 from Fig. 1 was utilized (here termed protocol 2b, see Fig. 4A). In this protocol, the 2,000×g step was added, aiming to separate ABs and MVs otherwise collected in the same pellet in the original protocol 2 (here termed protocol 2a, see Fig. 4A). The comparison of the RNA profiles showed that the rRNA peaks were most dominant in ABs and not in MVs from the HMC-1 and TF-1 cell lines. We can thus conclude that ABs are likely to contribute to a majority of the rRNA present in the pellet composed by a mixture of ABs and MVs (Fig. 4B).

### Different morphology of ABs, MVs and EXOs as visualized by TEM

**Fig. 5:**
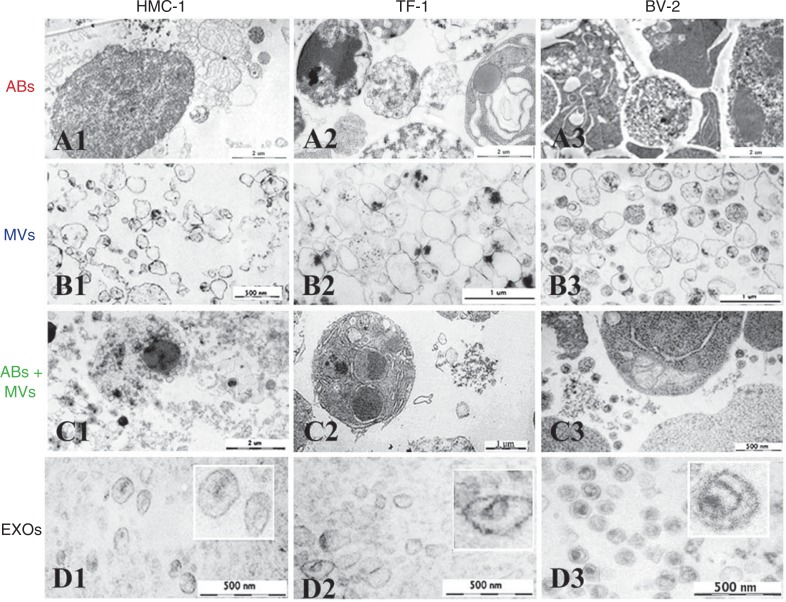
Analysis of ABs, MVs and EXOs by TEM. Micrographs of vesicles released from three different cell lines; HMC-1 (human mast cell line), TF-1 (human erythroleukemia cells), and BV-2 (mouse microglia cells) are shown. (A1–3) Dense structures show the chromatin substance in the generally round shaped apoptotic bodies (ABs) with a size of 800–5,000 nm. (B1–3) Microvesicles (MVs) are diverse in their shape and density, with a size range between 200 and 800 nm. (C1–3) In the pellet obtained by centrifugation at 16,500×g presents the mixture of ABs and MVs. (D1–3) The exosome (EXO) fraction from HMC-1 (D1), TF-1 (D2) and BV-2 (D3) cells were found to have a diameter of approximately 40–100 nm.

EVs containing pellets of HMC-1, TF-1 and BV-2 were visualized by TEM (Fig. 5). Images revealed that the pellet from the first step of centrifugation using protocol 1 (2,000×g) is composed by elements with chromatin condensation and/or marginalization with the size range of 800–5,000 nm that are characteristic of ABs (Fig. 5A1–A3). A very pure pellet was obtained from the second step (12,200×g) after 0.8 µm filtration. It contained predominantly round and oval shaped, membrane-bound structures of variable size and electron density within the diameter range of 200–800 nm (Fig. 5B1–B3). As expected, the first pellet obtained using protocol 2 (16,500×g) was composed by both ABs and MVs (Fig. 5C1–C3), as it contains elements with both 800–5,000 nm and 200–800 nm diameter, with typical characteristics of ABs and MVs. Finally, pellets from the last step of centrifugation (120,000×g) were composed of 40–100 nm diameter vesicles, consistent with EXOs (Fig. 5D1–D3).

The electron micrographs thus confirm that the respective pellets contain intact structures primarily within the expected diameter ranges and with typical morphological characteristics of the ABs, MVs and EXOs respectively. The images show similar morphology of the different vesicles from the three different cell lines analyzed, confirming that they produced similar subpopulation of vesicles isolated by the same centrifugation-based protocols.

### CD9, CD63 and CD81 are present on ABs, MVs and EXOs

**Fig. 6:**
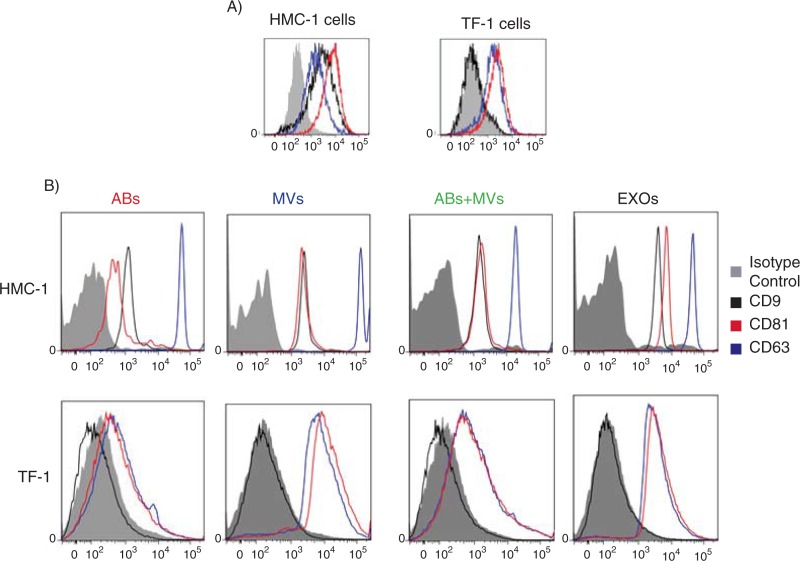
Detection and characterization of extracellular vesicles (EVs) by flow cytometry. The CD9, CD63 and CD81 expression on HMC-1 and TF-1 cells (A) and their expression on different vesicles, using anti-CD63-coated beads, are shown. (B) Cells and vesicles were immunostained against the tetraspanin (open curve) CD9 (in black), CD63 (in blue) and CD81 (in red) and compared with their appropriate isotype control (filled curve). The graphs are representative of n=3.

The presence of CD63 and CD81 on the surface of HMC-1 and TF-1 cells was confirmed by flow cytometry, whereas CD9 was only expressed on the HMC-1 cells, and not on the TF-1 cells (Fig. 6A).

In order to better characterize the different subpopulation of EVs, a flow cytometry-based evaluation of the tetraspanins CD9, CD63 and CD81 was performed (24). To do this, we utilized CD63-antibody-coated beads and could capture CD63-containing vesicles from all three types of vesicles (Fig. 6B) and not exclusively EXOs as previously suggested (28, 29). As expected based on flow cytometry results from cells, CD63-containing vesicles that were derived from HMC-1 cells, were positive for all markers investigated, whereas TF-1-derived vesicles bound to the CD63-antibody-coated beads expressed also CD81, as well as CD63, but not CD9 (Fig. 6B).

HMC-1 cells exposed less CD63 than CD81 at the cell surface (Fig. 6A), whereas conversely there was a higher level of CD63 than CD81 on the captured vesicles (Fig. 6B). TF-1 cells, instead, exposed both tetraspanins at the same level at their surface, and the released vesicles also expressed these two markers at the same level.

## Discussion

In this study, we have applied previously published centrifugation-based protocols considered appropriate for the isolation of ABs and MVs, respectively (22). Furthermore, we used protocols that are considered to remove ABs and MVs, and to more specifically isolate EXOs (21). These protocols were utilized to isolate the different vesicles from the supernatants of cultured HMC-1, TF-1 and BV-2 cells. Here, we provide evidence for the presence of clearly different RNA profiles in the various vesicle fractions, with rRNA being primarily detectable in ABs, and smaller RNAs without prominent rRNA peaks in EXOs. The isolates considered to be MVs contained little or no RNA, except for those from TF-1 cells. Indeed, electron microscopy of sectioned pellets of respective vesicle isolation revealed morphology compatible with predominantly ABs, MVs and EXOs in the different fractions, although some contamination between the fractions cannot be excluded. ABs and MVs were more diverse in their morphology than the EXOs. Flow cytometry revealed the presence of CD63 and CD81 positive vesicles in all fractions from all cell types, as well as CD9 except in the TF-1-derived vesicles.

For the separate isolation of ABs and MVs, we first used a 300×g centrifugation to remove cells, and a subsequent centrifugation with 2,000×g to pellet ABs. Subsequently, the supernatant was filtered and centrifuged at 12,200×g for the pelleting of MVs. Indeed, this approach separated vesicles containing rRNA, which was found primarily in the presumed fraction containing ABs, whereas no or little RNA was found in the MVs fraction, at least from the HMC-1 and BV-2 cells. The TF-1-derived vesicles showed a slightly different RNA profile, as rRNA profiles also could be seen in the MV fraction.

We do not know at this stage whether the rRNA identified in the TF-1-derived MVs fractions is indeed located in MVs, or it is present in other types of vesicles, or even in protein aggregates co-pelleting with MVs. However, electron microscopy of the pellets from the different vesicle isolations shows distinct morphological differences between ABs and MVs from all cells studied, which suggests that the rRNA in MVs from TF-1 cells is not necessarily due to a contamination from ABs, but may argue that MVs from some cells indeed does contain RNA.

With an approach previously utilized in our laboratory (21), a first centrifugation with 16,500×g was utilized after cell removal to collect ABs and MVs together prior to EXOs isolation. After that, EXOs were isolated by first passing the supernatant through a 200 nm filter, followed by 120,000×g ultracentrifugation. Thus, the first pellet contains both ABs as well as MVs, and the second pellet only EXOs. Using this approach, the RNA profiles of the mixed AB/MV pellet were similar to that seen in ABs using the first protocol, whereas the EXOs contain primarily small RNA without any prominent rRNA peaks (21). When a first centrifugation with 2,000×g was utilized prior to this protocol (to isolate ABs), again the rRNA profiles were in the HMC-1 cells seen in the AB fraction, whereas rRNA was found in both the AB and MV fractions from the TF-1 cell line. It cannot, however, be excluded that MVs from different cells have different capacity to carry RNA. Furthermore, it must also be considered that the RNA content of EVs may significantly change depending on the state of the cell (30). After 4 and 24 hours of TRAIL-induced apoptosis, increased concentration of rRNA was observed in the isolated ABs, compared to ABs from untreated cells. After 48 hours of TRAIL-induced apoptosis, a greater concentration of small RNAs was observed also in EXOs. This is in concordance with previously published data, showing that p53-induced apoptosis is associated with increased EXO secretion (31–33).

In many studies, flow cytometry with beads binding different types of micro- and nano-sized vesicles has been utilized (24, 28, 34), for example to determine the presence of the tetraspanins CD9, CD63 and CD81 on EXOs. Here, we have utilized beads with anti-CD63 antibodies, and could find positive signals in all vesicle fractions, except for CD9 in the TF-1-derived vesicles, which is not surprising, since TF-1 cells do not express this tetraspanin.

Comparing two kinds of cell lines analyzed, and the vesicles from them, HMC-1 cells express less CD63 than CD81 at the cell surface, whereas conversely there is lower level of CD81 than CD63 on the captured vesicles. This result could suggest that part of the CD63-vesicles may be CD81-negative and comes from intracellular compartments, whereas TF-1 cells expose both tetraspanins at the same level at its surface and the released vesicles also express these two markers at a similar level. These data indicate that the levels of CD63 and CD81 must be analyzed both at the surface of vesicle-secreting cells and in the resulting secreted vesicles. Results obtained using flow cytometry indicate that this method is not sufficient to establish that a nano-vesicle fraction studied is EXOs, and only EXOs. This may suggest that these surface molecules are not specific for EXOs, but are also present on ABs ad MVs from both HMC-1 and TF-1 cells. Alternatively, it may suggest that all three EVs population contain detectable amounts of CD63-positive EXOs. Importantly, in all flow cytometry analyses, events were also observed outside of the gates for the beads (data not shown), suggesting that a portion of vesicles do not bind to the CD63 beads, regardless of isolation protocol and vesicle fraction studied, again supporting the notion that non-CD63-expressing vesicles are present in all vesicular fractions.

The TEM analysis of the different vesicular fractions argues strongly that we primarily have ABs, MVs and EXOs in the different fractions from all the cells studied. Indeed, the morphology of all of these vesicles was characteristically similar to what previously had been described (22). Importantly, the contamination of, for example, EXOs in ABs and MVs is possible, but is not prominent according to the morphological characteristics.

This study shows that ABs, MVs and EXOs contain fundamentally different RNA profiles, and argues that MVs isolated from cell cultures often do not contain considerable amounts of RNA. The rRNA was primarily found in ABs, which should be considered when the functionality of RNA in different vesicles is studied.
